# Perspectives and experiences of parents of children with juvenile dermatomyositis: a semi-structured interview study

**DOI:** 10.1186/s12969-025-01079-2

**Published:** 2025-03-28

**Authors:** Amy Helen Kelly, Ayano Kelly, Davinder Singh-Grewal, Jeffrey Chaitow, Allison Jaure

**Affiliations:** 1 Department of Rheumatology, Campbelltown Hospital, Campbelltown, NSW Australia; 2 School of Clinical Medicine, University of New South Wales, South West Sydney Clinical Campuses, Liverpool, NSW Australia; 3 Department of Rheumatology, Liverpool Hospital, Liverpool, NSW Australia; 4 Ingham Institute of Medical Research, Liverpool, NSW Australia; 5 Department of Paediatric Rheumatology, Westmead Children’s Hospital, Westmead, NSW Australia; 6 Sydney School of Public Health, The University of Sydney, Sydney, NSW Australia; 7 Centre for Kidney Research, Westmead Children’s Hospital, Westmead, NSW Australia

**Keywords:** Juvenile dermatomyositis, Qualitative methods, Interviews, Patient-centred care, Caregivers

## Abstract

**Background:**

Juvenile Dermatomyositis (JDM) is a rare, childhood inflammatory disease and its management can be challenging and confronting for both clinicians and caregivers. Little is known about the perspectives of parental caregivers of children with JDM. This study aimed to describe the experiences of parents of children with JDM to inform person-centred care.

**Methods:**

Semi-structured interviews (face-to-face, telephone) were conducted with parents of children with JDM from three centres in Australia. Transcripts were analysed thematically.

**Results:**

Nineteen parents (15 mothers) of 17 children aged 8 to 21 with JDM participated. Six themes were identified. Rapid crescendo of fear and desperation (alarming deterioration, sudden realisation of seriousness, desperate for a diagnosis ), lost and unsupported in the health system (at the mercy of the medical team, frustrated at the lack of services, neglected priorities, protracted and painful search for answers), disrupting family routines (sibling neglect and loss, overloaded with a medicalised schedule, always on standby, burdened by financial strains), grieving what has been lost (missing the sunlight, struggling with the loss of physical function, disrupted schooling, changes in their child from steroid side effects), managing an uncertain future (bound to chronicity, fearing relapse, insecurity with transition to adult care), gaining confidence and motivation (strengthening partnerships with clinicians, growing maturity and independence, gaining hope from shared experiences).

**Conclusions:**

The diagnosis of JDM is often delayed and caregivers of children with JDM report distress, disruption and uncertainty throughout their treatment journey with their child. Addressing these fears and establishing support mechanisms that help parents navigate their way through the medical system and support changing family dynamics are vital to optimise health outcomes for children diagnosed with JDM.

**Supplementary Information:**

The online version contains supplementary material available at 10.1186/s12969-025-01079-2.

## Introduction

Juvenile Dermatomyositis (JDM) is a rare, chronic, childhood autoimmune disease, characterised by inflammation of small blood vessels of the tissues and organs, leading to a characteristic rash, muscle weakness, elevated muscle enzymes and sometimes involvement of vital organs with potential environmental triggers and genetic predisposition thought to play a role in its aetiology [1]. Better outcomes are achieved with early diagnosis and early aggressive treatment, often with significant side effects [2]. Due to the heterogeneity in presentation and the rarity of the disease, the diagnosis of JDM is often delayed [3], enhancing anxiety, confusion and a sense of isolation for caregivers of children with JDM.

Caregivers of children with JDM have reported increased levels of stress, higher levels of anxiety and poorer quality of life [3] and parenting stress may adversely affect child health-related outcomes as it could potentially interfere with the management of the child’s chronic illness [4]. The challenges for parents in caring for a child diagnosed with JDM may be amplified by the limited knowledge of JDM among non-paediatric rheumatology clinicians, difficulty accessing appropriate services, prolonged treatment, complexities in managing the condition, the burden of side effects of treatment and impacts on the family unit.

There are few studies examining the perspectives of parents or caregivers of children with JDM. This study aimed to describe the experiences of caregivers of children with JDM to inform strategies and interventions to address their needs.

## Methods

### Participant selection

Participants were eligible if they were a parent or caregiver of a child (aged 0 to 18 years) with clinician diagnosed JDM and were English speaking. Both parents were offered the opportunity to participate. Participants were recruited through the only public paediatric rheumatology centre in New South Wales, Australia, the Sydney Children’s Hospital Network, which includes paediatric rheumatology clinics at The Children’s Hospital Westmead, The Children’s Hospital at Randwick and John Hunter Hospitals. Potential participants were identified by the paediatric rheumatology team as a family with a child diagnosed with JDM. AHK independently approached the family after they had received an introductory letter to the study from their treating team. AHK had no involvement with their child’s clinical care. Purposive sampling was used to capture a broad range of perspectives based on socioeconomic status, geographic location, ethnicity, their child’s disease course, sex and age. All participants consented to de-identified data being recorded and transcribed and included in the final paper. The project was approved by the Sydney Childrens’ Hospital Network Ethics Committee under project number 2021/ETH00053.

### Data collection

A preliminary interview guide was developed based on the literature of the experiences and perspectives of caregivers of children with other paediatric rheumatic diseases [3, 5, 6]. AHK conducted a semi-structured interview, approximately 40 minutes in duration, either face to face at a hospital clinic or by telephone, depending on participants preference or COVID- 19 requirements. Recruitment ceased once data saturation was reached (when no new themes or new concepts were emerging in the data). Interviews were recorded and then transcribed verbatim.

### Data analysis

Transcripts were entered into HyperRESEARCH version 4.0 to assist with the coding, storage and searching of the data. Using thematic analysis, as described by Braun and Clark (2006) [7] the first author (AHK) coded the transcripts, line by line, conceptualising and categorizing the data and assigning codes to inductively identified concepts. Relationships between common concepts were explored in the data to develop analytical themes, according to Braun and Clark’s [7] definition of thematic analysis, where a theme captures *something important about the data in relation to the research question*, *and represents some level of patterned response or meaning within the data set* [7]. A thematic schema was mapped to demonstrate the connection between themes. A second and third investigator AJ and AK, read and reviewed the preliminary themes to ensure that all experiences and perspectives of participants were included. AHK identified quotes that best captured the themes and AK and AJ reviewed the quotes and consensus was reached as to their appropriateness. All participants consented to de-identified quotes from their interviews being included in the final paper.

## Results

The characteristics of the participants are shown in Table 1. Nineteen of 27 (70%) caregivers who were contacted agreed to participate. The majority of interviews were conducted via telephone (*n* = 13, 72%). The majority of participants were female (15, 79%) and all participants identified as their child’s biological parent. Table 2 details the characteristics of the children of participants, including their initial presentation, treatments they received, duration of their illness at the time of interview and the number of specialists they had seen prior to a diagnosis being made.

**Table 1:** Characteristics of participants

Characteristic	No. (%)
Biological Mother	15(79)
Biological Father	4(21)
**Age (years)**	
40s	14(74)
50s	4(21)
60 and over	1(5)
**Education**	
Secondary	1(5)
Certificate/Diploma	2(11)
Bachelors/Higher	16(84)
**Marital Status**	
Married/defacto	19(100)
**Employment**	
Casual	1(5)
Full time	9(47)
Part time	5(26)
none	4(21)
**Geographical Location**	
Metropolitan	14(74)
Rural	5(26)
**Religion**	
Religious affiliation	10(53)
**Ethnicity**	
Caucasian	15(79)
Greek	1(5)
Fijian Indian	1(5)
Japanese	2(11)

**Table 2:** Characteristics of the children of participants

Characteristics	No. (%) *n* = 17
Females Males	9 (53) 8 (47)
Country of birth	
Australia Other	16 (94) 1 (6)
Religion	
Religious affiliation	10 (59)
Ethnicity	
Caucasian/Australian CALD	13 (76) 4 (24)
Clinical presentation of illness	
Muscle weakness Skin rash Lethargy Pain Falls	8 (47) 11 (65) 1 (6) 4 (24) 2 (12)
Age at diagnosis	
1–5 years 6–11 years 12–15 years 15–20 years	8 (47) 4 (24) 5 (29) 0 (0)
Current treatment	
Prednisone Methotrexate IVIG Other DMARD Tofacitinib None	6 (33) 11 (61) 3 (17) 1 (5) 2 (11) 4 (22)
Previous treatment	
None Methylprednisone Prednisone IVIG Methotrexate Other DMARD	3 (17) 4 (22) 10 (56) 1 (5) 7 (39) 1 (5)
Comorbidities	
None Perthes disease Asthma Coeliac disease ADHD Osteoporosis Skin striae	11 (61) 1 (5) 1 (5) 1 (5) 1 (5) 1 (5) 2 (11)
No. of doctors initially referred to	
1 2 3 4	4 (22) 11(61) 2(11) 1(6)
Duration of illness (years)	
1–2	7 (40)
3–5	7 (40)
6–10	3 (20)

**Fig. 1:**
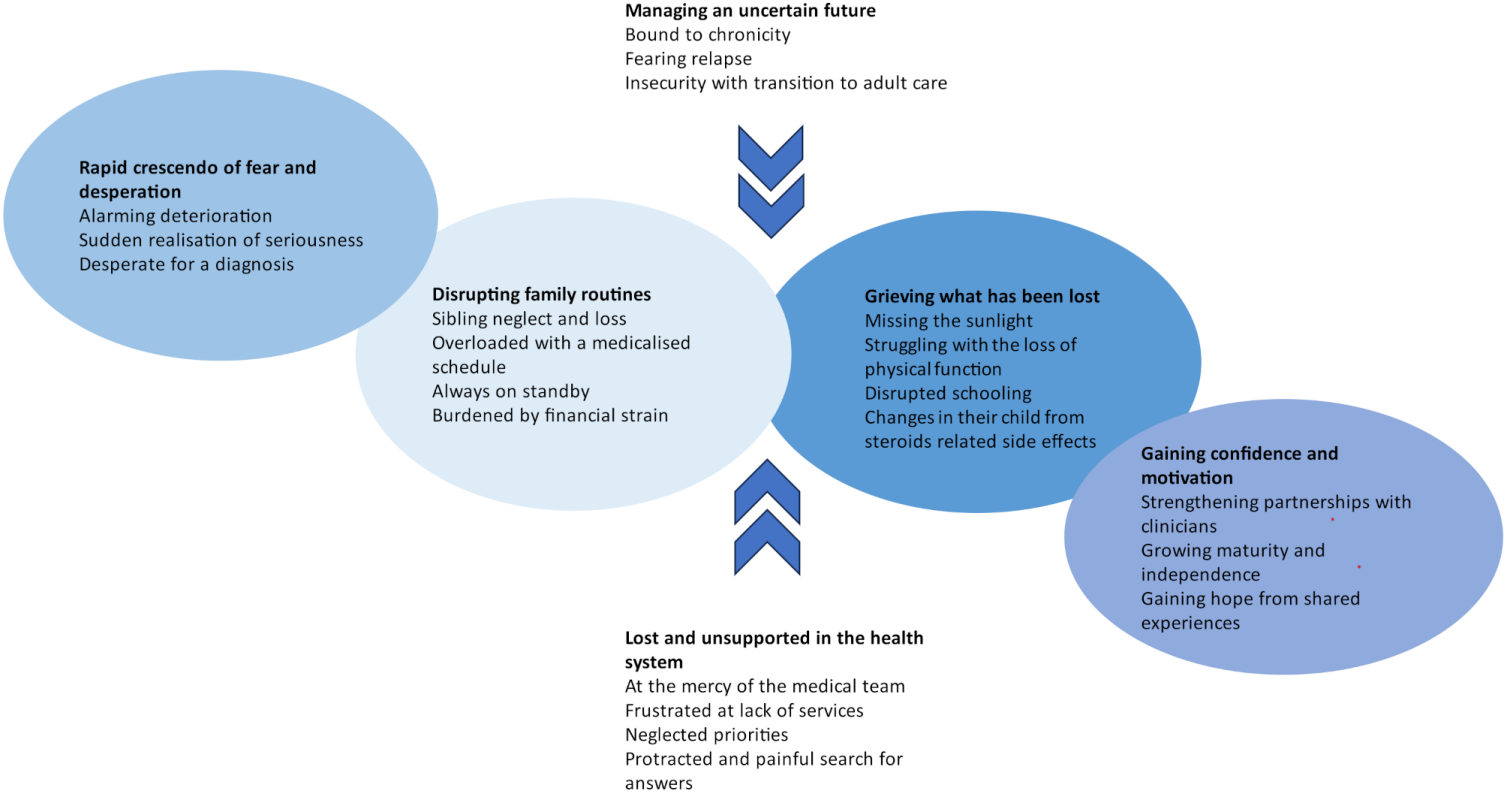
Schematic diagram of themes and subthemes. Rapid crescendo of fear and desperation disrupted family routines, which contributed to parents’ grief at the loss of their normal family life. Feeling lost and unsupported in the medical system and managing an uncertain future also contributed to disrupted family routines and parents grieving what they had lost. Parents fears, struggles with services, changes in family routine, navigating a complicated health system, over time, evolved into them gaining confidence and motivation

We identified six themes: Rapid crescendo of fear and desperation, lost and unsupported in the health system, disrupting family routines, grieving what has been lost, managing an uncertain future, gaining confidence and motivation. The respective subthemes are described below and selected quotations are illustrated in Table 3. Figure 1 illustrates the relationship between these themes in a thematic schema.

**Table 3:** Selected quotations supporting each theme

Themes and Subthemes	Quotations
**Rapid crescendo of fear and desperation**	
Alarming deterioration	*She never goes to the doctor because she’s never sick*, *and then one day she said to me ‘can you take me to the doctor*, *it’s really hurting’*, *and that’s when I thought oh*, *God*, *this must be not growing pains. (mother).*
Sudden realisation of seriousness	*…she needed help wiping herself and things like that*, *and we were like oh God*, *what’s going to happen*, *are we going to have to become carers*, *are we going to have to*, *one of us resign our jobs*, *things like that. (mother).*
Desperate for a diagnosis	*…then we couldn’t get him to see the neurologist for I think two months. We had this terrible time where we didn’t know what it was and we didn’t know what to do. (mother).*
**Lost and unsupported in the health system**	
At the mercy of the medical team	*I do feel a little in the dark….sometimes I wonder if there are things we are just not being told (father).*
Frustrated at lack of services	*Because JDM is so specialised*, *you didn’t really fit anywhere (mother).*
Neglected priorities	*Having a teenager is pretty stressful as you may know…but the combination of a teenager and then someone who’s unwell*, *the compounding can impact mental health (mother).*
Protracted and painful search for answers	*We probably saw six or seven doctors. Only one had heard of it*, *but didn’t know much about it (father)*
**Disrupting family routines**	
Sibling neglect and loss	*…during that time*, *she basically lost a little friend…That was emotionally quite difficult for her and obviously for him*, *because he just wasn’t able to do anything (mother).*
Overloaded with a medicalised schedule	*Generally*, *you find that when you come into the hospital and you see the rheumatologist*, *it’s busy. It’s chaos…I’d like to be able to ask a question and have a bit more time (mother).* *I guess the medication is probably the biggest responsibility*, *just ensuring that that’s all okay and making sure his scripts*, *monitoring that (mother)*
Always on standby	*If he needs to take a day off school because he’s just not up to it*, *you know*, *I make sure I work from home that day…(father).*
Burdened by financial strains	*Our family was based on my single income and (my wife) wasn’t working at the time. I just had to find a way to somehow keep it together (father).*
**Grieving what has been lost**	
Missing the sunlight	*I know she shouldn’t be out in the sun*, *and I know that that’s not great*, *but I don’t know to what extent…I was like*, *does that mean no lunch time at school*, *no sport? (mother).*
Struggling with the loss of physical function	*…he was walking with someone who had a broken arm*, *and he was feeling just as sick as this kid that had a broken arm*, *and people were saying to him ‘well*, *why aren’t you helping that boy with the broken arm*, *because you’re fine?’ and he was like I’m not fine*, *I’m really struggling to walk up and down the stairs. I think because you can’t see it*, *people are not as empathetic (mother).*
Disrupted schooling	*… there are days when he’s got a headache or a cold now… I’m a bit more lenient. If he needs a day off here or there*, *I’m all right (mother).*
Changes in their child from steroid related side effects	*Once he started the prednisone it completely interrupted his sleep*, *so there’s some days he’s completely exhausted from waking up continuously through the night (mother).*
**Managing an uncertain future**	
Bound to chronicity	*…what’s it going to do*, *will it go away*, *will it get worse? You know…it’s a very unpredictable disease*, *it can be chronic (mother).*
Fearing relapse	*I think it’s always there and present for him*, *because he knows it will never go away. So yes*, *I think maybe that’s the uncertainty around the disease coming back (mother).*
Insecurity with transition to adult care	*The longer-term impacts for that person with JDM*, *because I believe the JDM will always be a juvenile autoimmune related condition*, *it won’t move into an adult type– it may or may not*, *but it’s always going to be treated like a JDM. Just understanding maybe a little bit more about that in an adult*, *or that transition to adulthood. Maybe that’s what could be improved*, *actually*, *that whole transition to the adult world (mother).*
**Gaining confidence and motivation**	
Strengthening partnership with clinicians	*They relied on our observation to report anything that’s happening on a day to day basis and how the treatment is going. We felt quite involved in the treatment in that way*, *which was good (father)*
Growing maturity and independence	*He is conscious of eating properly now. Whether this is due to the experiences that he had with this supposed dietary intervention or whether he’s doing it because he just doesn’t want to appear overweight*, *he’s conscious about what he eats. I think it’s in a positive space at the moment (father)*
Gaining hope from shared experiences	*But she’s scoring goals*, *she’s getting awards*, *she’s loving it. Socially*, *she’s loving it*, *and the teams are fantastic. That’s one of the joys now that we’re out the other end (father).*

### Rapid crescendo of fear and desperation

#### Alarming deterioration

Parents felt frightened and panicked at the rapid decline they saw in their child’s physical abilities. They were very concerned to witness their child being unable to do up their seat belt, falling over at the supermarket, or being unable to get out of bed, *“… we went away bushwalking with some friends*, *and I had to carry her in the backpack”* (mother).

#### Sudden realisation of seriousness

Parents initially were dismissive of child’s symptoms as a minor illness, part of normal development, or even growing pains, *“I just thought it might be a cold. I just thought it was growing pains.”* (mother). However, they gradually began to realise the seriousness of their condition as they could see their child was not recovering and, in many cases, deteriorating.

#### Desperate for a diagnosis

Parents felt an increasing desperation when numerous clinicians were unable to find a diagnosis for their child’s symptoms *– “The hardest part was the long wait to diagnosis”* (mother). They felt disappointed and let down because of the delayed diagnosis, particularly when they felt general practitioners did not know about the condition– “*our GP had not even heard of it”* (mother). They found it difficult to understand that there was no clear answer as to why their child developed JDM and described feeling stupid when they repeatedly asked the clinicians why this happened to their child. They felt a sense of relief once the diagnosis of JDM was made.

### Lost and unsupported in the health system

#### At the mercy of the medical team

Parents felt intimidated and disempowered by the use of medical terms that were difficult to understand. Protocols for administration of medications, including weekly dosing and the regular blood monitoring for medications also contributed to a sense of being overwhelmed with a rigid medical schedule. Parents relied on the medical team to educate them about JDM and its treatments. However, many parents described feeling lost, “*I do feel a little in the dark…. sometimes I wonder if there are things we are just not being told”* (father). Parents from culturally and linguistically diverse backgrounds (CALD), who were not familiar with the health system found it even more difficult to understand. Parents felt overwhelmed by the complexity of the disease including the varying way the disease could initially present and its response to treatment. They understood that treatments were being determined by an individual clinician’s experience, leading them to feel like there was no standard treatment protocol being followed, ‘’*There seems to be so much happening with the disease… it’s all very subjective*, *I feel like we are 100% reliant on the* (Doctor’s) *gut feel”* (mother).

#### Frustrated at lack of services

Parents were shocked by the limited services once their child was diagnosed with JDM. They described feeling forgotten and alone when they discovered there was no single point of contact to coordinate their child’s care. Parents in rural or remote areas felt vulnerable as it was difficult to travel long distances to medical appointments in a large metropolitan city, *“it was scary being 5 hours away from the specialist*, *it’s hard when we are the only ones in a rural town* (with the disease)’’ (mother).

#### Neglected priorities

Parents reported being disillusioned and upset when they perceived that clinicians were focussed on the treatment of muscle weakness and did not address other symptoms, including pain, and mental health issues, *“I can’t localise it*, *it’s pretty broad. It’s either muscle pain*, *back pain*, *stomach pain*, *it comes in different forms. I don’t think that has really been addressed or understood in a detailed manner….it’s something that should’ve been teased out a little bit more” (father).*

#### Protracted and painful search for answers

Parents were desperate for the treatments for their children to work, they were prepared to try anything, and some sought non-pharmacological ways to manage the condition including seeking out alternative therapies “*We did try a few elimination diets”* (mother) and, “*We were prepared to try anything” (mother)*, knowing that these were not recommended therapies for children with JDM. Parents described spending many hours researching online and struggled with the lack of specific information relevant to the Australian health care system. Parents discovered that treatment practices varied internationally depending on the availability of treatments and the local health care system. Variation in treatment practices internationally coupled with discovering worst-case scenarios online, including finding examples of children with lifelong disabilities, was both alarming and frightening.

### Disrupting family routines

#### Sibling neglect and loss

In focussing their attention on their child with JDM, some parents felt that their relationship with their other children, or among siblings, suffered. For example, previously siblings were able to play together, this was not possible because of disease symptoms or medication side effects, ‘’*she basically lost a little friend’* (mother). Participants were frustrated that their other children were missing out on school and important life events because of the time taken caring for their child with JDM. Parents were also concerned for future potential mental health problems for siblings, *“we* [the whole family, including siblings] *were semi-suicidal for a lot of last year…”* (mother).

#### Overloaded with a medicalised schedule

Attending multiple medical appointments was time consuming and parents described the changes they had made to their family’s routine ‘’*our family has made Saturday a rest day; that is the day she takes medications’’* (mother). Parents spoke of having to accommodate the burden of responsibility for managing their child’s medications, including minimising family activities because of side effects from medications, monitoring for their child’s response to treatment as well as being the central coordinators of their child’s care, made them feel that they were different to other families; *“we are not a normal family anymore’’* (mother).

#### Always on standby

Some participants described having to give up all other parts of their lives, including their job, travel, social events and career aspirations to constantly be there for their child, *‘’I sat outside the school for hours*, *knowing that he might need me to come and get him at any point”* (mother). This was described as frustrating, emotionally demanding and time consuming, particularly for mothers who felt they carried the majority of the caring load for their child.

#### Burdened by financial strains

Parents were stressed juggling their work or business, for example, time taken attending multiple medical appointments was time they had to take off work, which was particularly burdensome when they ran their own businesses. Families carried the burden of costs of travelling to multiple appointments (particularly for regional families), including self-funding allied health therapies, because there were no publicly available services. Finances were a source of tension in many households, “*the only thing we fight about every month is the credit card bill”* (mother).

### Grieving what has been lost

#### Missing the sunlight

Parents understood it was important to avoid the sun to prevent worsening their child’s skin disease. It saddened them to have to reconsider family outings and avoid the hottest time of the day, however it also provided an opportunity to proactively manage part of their child’s disease. Sun exposure did cause anxiety in parents, and they were confused and worried about managing their child’s exposure to the sun particularly when they were not with them. They felt sorry for their child when they missed out on activities to avoid the sun; “*You’ve got a little boy who just wants to be like every other kid and doesn’t want to be out of the sun*” (father).

#### Struggling with the loss of physical function

Parents were upset at the manifestations of the disease in their child and felt compelled to protect their children from the negative comments of others, knowing that their child’s symptoms were only visible to those that knew them well. They felt vulnerable and defensive towards those that did not appreciate the suffering of their child, ‘’*He was walking with someone who had a broken arm*, *and he was feeling just as sick as the kid with the broken arm and people were saying*, *why aren’t you helping that boy with the broken arm?”* (mother). Parents wanted their child to do more, hoping that exercise would improve their symptoms, and they felt guilty when they saw their child struggling or in pain.

#### Disrupted schooling

Parents were despondent to see their child struggle to attend school because of their illness and suffer from side effects of treatment; “*my child is a primary school drop out’’* (mother). The majority of parents felt supported by the child’s school, however some reported having to justify and negotiate more support services from their school, “*school says he will catch up….but this disease isn’t going anywhere”* (mother). Paradoxically, parents reported that COVID remote learning helped their child focus on improving their health whilst maintaining connection with their schoolwork, in a safe environment, ‘’*We had a COVID safe bubble*” (mother). Parents described a sense of relief and satisfaction that their child was not missing out on school.

#### Changes in their child from steroid related side effects

Participants described feeling very despondent by the changes they saw in their child from the side effects of treatments, particularly steroid side effects. They saw their child gain weight and become self-conscious *‘’every couple of months we go up a size*, *he is starting to feel different to other kids”* (mother). Parents were upset by memories of their child’s struggles and were sensitive to their self-image, which was amplified when their child moved into their teenage years, *“’She won’t even go back and look at photos of herself when she was like that* (mother). Other steroid side effects, including emotional lability was very alarming; “*I give my son toxins every day that make him feel like he wants to die*, *and then have to spend the rest of the day telling him that it’s worth living and there is hope”* (mother).

### Managing an uncertain future

#### Bound to chronicity

Parents did not expect JDM to take so long to achieve remission, nor did they initially appreciate the need for ongoing treatment, “*we didn’t realise that it’s not fixed overnight’’* (mother). Parents became aware that their child may cycle in and out of remission and many parents were shocked and confused by this, they had not expected the disease to behave in this way.

#### Fearing relapse

Participants described their greatest fear was a relapse of their child’s illness, requiring further treatment and the potential side effects that may follow. They felt anxious about what the future might hold for their family; to the extent that some “*tried not to think about the future”* (mother). Parents were unsure as to how they or their child would cope if they experienced a relapse of the disease.

#### Insecurity with transition to adult care

Parents were concerned about how their child would transition from paediatric care to adult medical services, including worrying about how their child would navigate the complexity of their disease such as sun avoidance and know if their disease had relapsed,… *I think the transition to adult and the adult rheumatology area*, *that’s probably where we’re a bit nervous at the moment because we know next year when he finishes school*, *he has to move into the adult world* (mother).

### Gaining confidence and motivation

#### Strengthening partnerships with clinicians

Parents reported wanting to know about the objective markers of their child’s disease and to understand if there had been improvements with treatment. *If there was more of a… some type of tracking system where we could put in how we think the skin’s doing*, *blood results*, *those types of things could be monitored all together. Rather than just having a look at him every couple of months. I think that would really help with our anxiety around what is happening* (father).

Parents appreciated feeling involved in their child’s treatment. This was aided by clinicians sharing pathology results with them, such as creatinine kinase (CK) to monitor response to treatment. At regular follow up appointments, they waited anxiously for their child’s results and celebrated when they saw improvements in objective markers, such as CK, “*We always looked at the CK*’’ (father).

#### Growing maturity and independence

With the intense focus on their child’s physical health and managing side effects, such as weight gain from steroids or worsening skin disease from sun exposure, parents saw their child become more aware of healthy habits and avoiding sunlight. Parents felt a sense of pride when they observed how their child matured and learned to navigate their condition with their support, “*I know that [he has an] awareness that he doesn’t want in be in the sun”* (mother).

#### Gaining hope from shared experiences

Parents found it helpful to talk to other parents affected by JDM, they wanted to understand other’s experiences. This enabled them to visualise what disease remission looked like. It was their way “…. of *looking for the light at the end of the tunnel’’* (father), and it gave them hope for the future.

## Discussion

Previous studies have indicated that parents caring for children with JDM experiences higher rates of psychological disturbance including anxiety and emotional distress [8]. Our study confirmed similar findings. In our study parents of children with JDM experienced a sudden fear when they realised that their child was seriously unwell and become desperate for a diagnosis. They felt frustrated at the lack of knowledge by many in the medical community about JDM. Once the diagnosis had been made and their child was receiving treatment for JDM, parents described feeling overwhelmed by the rigid medical schedule that took over their family life. They felt isolated because of the lack of services to support their child, finding it challenging to access allied health services with adequate knowledge of the disease. Parents found it difficult to process the diagnosis because their doctors were unable to determine the cause of JDM. Parents also reported experiencing increased financial strain. Complicated treatment regimes, including high doses of steroids, sun avoidance measures and the requirement for regular blood monitoring led parents to describe a sense of feeling overwhelmed by the “medicalisation’’ of their lives and they grieved the disruption in their family’s routine. Parents were saddened to see their child have their schooling disrupted and feared relapse, often because they had seen online worse case scenarios of children with severe disease, yet they gained hope and confidence when they witnessed positive progress in their child. Parents were pleased when they saw the development of independence and confidence in their child’s self-management.

Parents of children with chronic illness experience greater levels of stress and lower quality of life [3]. A study examining the experiences of caregivers of children with JDM in the United States, reported that parents’ quality of life was reduced and their mood adversely affected [3]. Our study identified similar stress amongst parents. Another childhood rheumatic disease, Juvenile idiopathic arthritis has been described as a family disease [6], similarly our study also identifies direct impacts on parents and their families. Parents described their sadness at the disruption to their normal family routine, changes in relationships between siblings, demands of attending medical appointments and the need to drop everything to support their child. These burdens were especially carried by mothers in our cohort and existing evidence in the literature confirms the major caregiving role to the mother [9] in childhood chronic diseases. The demands of caregiving and effects on financial resources can have a negative impact on women’s professional and social lives [9].

Our study provides broad insights into the perspectives of parents of children with JDM. Purposive sampling was used to include a diverse group of participants including men, women, culturally and linguistically diverse participants and those from regional and metropolitan areas, however, there are some potential limitations. Most of the participants were mothers with a high level of educational attainment, aged in their 40’s or over and only approximately 20% of participants were from culturally and linguistically diverse backgrounds (CALD). The transferability of our findings to fathers, young parents, less educated or non-English speaking populations is thus uncertain. We acknowledge that we may have recruited those individuals highly motivated to participate in research, which may bring some inherent bias into the study. We note however that there was reasonable diversity in the duration of the children’s disease duration at the time of interview (see Table 2), which may have documented the perspectives of parents with years of experience versus those with newly diagnosed children. Further limitations to our study include that we may not have captured an accurate representation of children’s experiences of their disease, by interviewing their parents. Previous studies have suggested that using parents as a “proxy” for their child’s experience may underestimate the child’s experience, specifically regarding health-related quality of life [11]. The majority of the interviews were via telephone (*n* = 13) and it is acknowledged that this may have influenced participants responses to the questions asked.

We identified specific areas of concern for parents with implications for clinical practice, including delayed diagnosis, lack of JDM specific services, monitoring treatment response, sun avoidance strategies and disruption to schooling. Limited awareness of JDM, its heterogenous presentation [5] and limited paediatric rheumatology services across Australia all contribute to the likelihood of a delayed diagnosis. Previous studies have indicated that the mean time to diagnosis is 8.5 months [3]. In our cohort, there was a high likelihood of delayed diagnosis, with 78% of participants reported seeing at least 2 specialists (after being referred by their General Practitioner) prior to the diagnosis of JDM being made (Table 2). In other chronic diseases, delayed access to specialist care results in deteriorating health, more frequent hospital admissions and poorer health outcomes [10]. This is particularly true for rural and regional patients, who may have to travel long distances with the associated time and the financial demands that entails [10]. It may not be possible to educate every health practitioner specifically about JDM, however understanding abnormal musculoskeletal and/or dermatological presentations in children is integral to reducing the time to diagnosis, in addition to the urgent need to expand access to paediatric rheumatology services across Australia.

We identified further implications for clinical practice, including treating teams recognising the importance of involving parents and children in their disease’s management. Parents felt a sense of success [12] when “celebrating” a normal creatinine kinase level or seeing improvement in their child’s CMAS (Childhood Myositis Assessment Score). Many reported that closely following sun avoidance recommendations disrupted family routines, however managing their child’s sun exposure also gave parents a sense of control over an aspect of their child’s disease. Clinicians should focus on effectively communicating the goals and measurements of treatment, recognising the importance of communicating when those goals have been met and providing a clear framework for sun avoidance measures, to avoid confusion and empower parents. Further, parents understand the importance of minimising disruption to their child’s schooling [13], with current research suggesting that school disruptions during the COVID-19 era had adverse effects on child health and wellbeing [14]. A previous study suggested that families of children with JDM were worried and anxious during the COVID-19 pandemic relating to disruptions in their treatment or isolation from their school and usual support structures [15]. Our study overlapped with the COVID-19 pandemic and participants reported a sense of relief that their child was not missing out on educational opportunities when their school was in lockdown, when compared to their peers. Multidisciplinary management of families with JDM could therefore include working more collaboratively with educational partners to ensure minimal disruption to schooling. Pandemic home-schooling models may provide a template for JDM patients to better engage in schoolwork.

Our study identified rationalising the use of steroids as an important area for future research. In our cohort, parental stress was impacted by steroid related side effects. Rheumatic diseases are often complex in their management and often require long term management [16]. Similarly JDM may also require complex medication regimes, often lacking evidence base and usually relying on the experience of the treating paediatric rheumatologist [17]. It may be difficult to predict an individual patient’s response to treatment [2] and high dose steroids may be required [18], with the potential for significant side effects including; weight gain, stunted growth, bone loss [19], mood disorders and psychosis [20]. Children have reported they often fear the side effects of medications such as steroids and methotrexate [11]. The distress and morbidity caused by side effects of high dose steroids (which can in part fuel the fear of relapse for parents) highlights the urgency to find new, less toxic treatments.

## Conclusion

Our study provides a unique insight into the experience of caregivers of children with juvenile dermatomyositis as they navigated their child’s initial presentation, diagnosis and response to treatment. Key areas of concern include delays in diagnosis, lack of access to services, response to treatment, fear of relapse, toxic side effects from medications and the unknown causes of the disease. This study provides an important platform for understanding how to better support families with JDM, providing clinicians and policy makers the evidence they need to improve the comprehensive management of this rare disease, to enhance treatment strategies and better inform future research directions.

## Electronic supplementary material

Below is the link to the electronic supplementary material.

Supplementary Material 1

## Data Availability

The datasets used and/or analysed during the current study are available from the corresponding author on reasonable request. Competing interests: The authors declare that they have no competing interests.

## Abbreviations

JDM: Juvenile Dermatomyositis
AHK: Dr Amy H Kelly
AJ: Professor Allison Jaure
AK: Dr Ayano Kelly
CK: Creatinine Kinase
CMAS: Childhood Myositis Assessment Score
CALD: Culturally and linguistically diverse
