# Supplementary material for: Perspectives and experiences of parents of children with juvenile dermatomyositis: a semi-structured interview study
Source: Pediatr Rheumatol Online J. 2025 Mar 28;23:34. doi: 10.1186/s12969-025-01079-2 (PMC11951517; doi:10.1186/s12969-025-01079-2)
Supplement: Supplementary file 1 — Supplementary Material 1 [file 12969_2025_1079_MOESM1_ESM.docx]

**Appendix: *Interview guide for Perspectives and experiences of parents of children with juvenile dermatomyositis: a semi structured interview study.***

1. **Introduction**

• Explanation of the study, confidentiality, obtain informed consent, questions.

• Can you tell me about how you first found out your child had JDM?

• Thinking from the time your child was diagnosed up until now, what areas of your life have been most impacted by JDM; what has been most challenging - why?

1. **Personal and role**

- In what way has caring for a child with JDM impacted you – personally?
- What are the added responsibilities and do you feel about these/cope with these? (medications, school)

1. **Relationships (family/social) and financial**

- Has it impacted on your spouse/family – in what way and how do you cope with this? (relationships)
- Did it have any impact financially – in what way?

1. **Healthcare and treatment management**

- On a scale of 1-10 (where 1 is not confident at all and 10 is very confident) how confident do you feel about managing your child’s health/treatment – why?
- How accessible are health care services for you and your child?
- How involved do you feel you are in making treatment decisions? Can you give some examples? *(access, communication)*
- How would you describe your relationship with your child’s health care team?
- Are there any other support groups or alternative health care services that you access?

1. **Outlook and prognosis**

- What do you consider to be the long term outlook for your child– challenges? *(health, career, education, social)*

1. **Support**

- Do you get support from family/school/community? – details
- How could support be improved for parents caring for a child with JDM
- What kind of information or support do you think is important for families living with a child diagnosed with JDM?
- What advice would you give to families who have just found out their child has JDM?

1. **Close:** Is there something else that you think is important to add?
